# Elastic Critical Moment for the Lateral–Torsional Buckling (LTB) Analysis of Structural Glass Beams with Discrete Mechanical Lateral Restraints

**DOI:** 10.3390/ma13112492

**Published:** 2020-05-29

**Authors:** Dario Santo, Silvana Mattei, Chiara Bedon

**Affiliations:** Department of Engineering and Architecture, University of Trieste, 34127 Trieste, Italy; dario.santo.ds@gmail.com (D.S.); silvana.mattei@phd.units.it (S.M.)

**Keywords:** structural glass beams, laminated glass sections, lateral–torsional buckling (LTB), discrete mechanical lateral restraints, analytical methods, finite element (FE) numerical modeling, design

## Abstract

Structural glass beams and fins are largely used in buildings, in the form of primary load-bearing members and bracing systems for roof or facade panels. Several loading and boundary conditions can be efficiently solved by means of bonded composites that involve the use of laminated glass sections. Additionally, the so-obtained glass members are often characterized by high slenderness. To this aim, several literature studies were dedicated to the lateral–torsional buckling (LTB) behavior of laterally unrestrained (LU) glass elements, with the support of full-scale experiments, analytical models, or finite element (FE) numerical investigations. Standardized design recommendations for LU glass members in LTB are available for designers. However, several design issues still require “ad hoc” (and often expensive) calculation studies. In most of the cases, for example, the mechanical interaction between the structural components to verify involves various typologies of joints, including continuous sealant connections, mechanical point fixings, or hybrid solutions. As a result, an accurate estimation of the theoretical LTB critical moment for such a kind of laterally restrained (LR) element represents a first key issue toward the definition and calibration of generalized design recommendations. Careful consideration should be spent for the description of the intrinsic features of materials in use, as well as for a combination of geometrical and mechanical aspects (i.e., geometry, number, position of restraints, etc.). In this paper, the attention is focused on the calculation of the elastic critical buckling moment of LR glass beams in LTB. Existing analytical approaches of the literature (mostly developed for steel constructional members) are briefly recalled. An additional advantage for extended parametric calculations is then taken from finite element (FE) numerical analyses, which are performed via the LTBeam or the ABAQUS software codes. The actual role and the effect of discrete mechanical restraints are, thus, explored for selected configurations of practical interest. Finally, the reliability of simplified calculation approaches is assessed.

## 1. Introduction and State of the Art

Structural glass is largely used in building, in the form of load-bearing components [1]. While harmonized European standards for structural designs are still in preparation [2,3,4], the last few years showed a huge spread of technical guidelines, codes of practice, and documents in support of designers [5,6,7].

**Figure 1:**
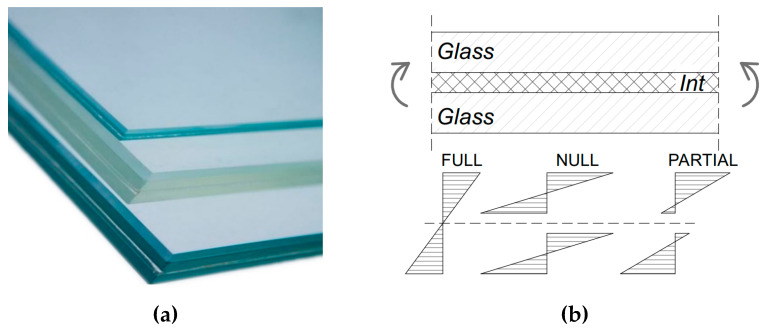
Laminated glass element: (**a**) cross-section examples and (**b**) corresponding stress distributions, depending on the shear coupling efficiency of the interlayers.

For structural applications, glass members are typically characterized by a laminated resisting cross-section, in which viscoelastic bonding interlayers are required to offer a certain mechanical coupling to the involved glass components (Figure 1a). Accordingly, for a given geometry, the load-bearing response of glass layers can vary significantly (Figure 1b). Among the available interlayers [8], it is in fact generally recognized that that the mechanical response of a general laminated section is strictly related to the actual properties and bonding efficiency of these films [9,10]. As a result, refined methods of analysis and characterization should be generally taken into account, in the elastic stage [10,11,12] or in presence of possible degradation effects [13,14,15].

**Figure 2:**
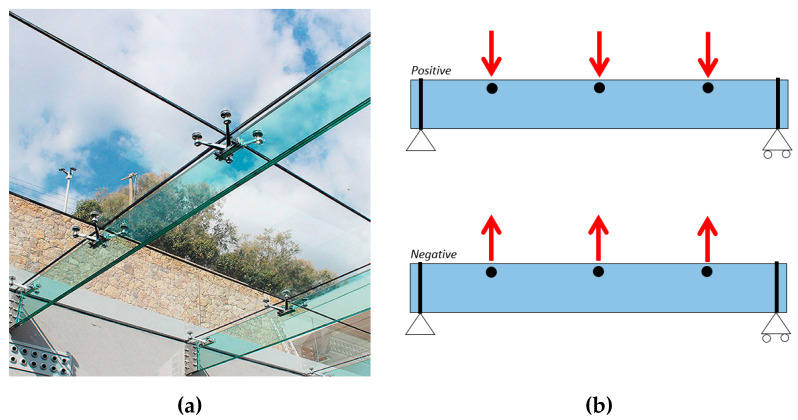
Example of (**a**) laterally restrained (LR) glass beams with discrete mechanical restraints and (**b**) typical loading configurations associated with positive or negative bending effects.

As far as glass beam-like elements are used to support and brace orthogonal facades or roofs, careful consideration in design should be spent to prevent premature lateral–torsional buckling (LTB) phenomena. Several research studies, in this regard, were dedicated to monolithic or laminated glass beams. Extended experimental investigations were reported in [16,17,18,19,20,21] for various LTB configurations of practical interest. The use of simplified, equivalent thickness-based formulations for laminated glass sections in LTB was explored in [22], toward the definition of reliable and standardized design buckling verification approaches [23,24,25,26]. These documents and most of the literature efforts, however, are generally focused on laterally unrestrained (LU) glass beams that roughly represent the actual load-bearing configuration of real fins (i.e., Figure 2). 

A limited number of background documents is currently available for laterally restrained (LR) glass members in LTB that can take advantage of the presence of possible bracing contributions, toward premature failure mechanisms. According to Figure 2a, for example, mechanical restraints can be used for fork-end supported, laminated glass fins acting as a bracing system for the interconnected orthogonal panels. The typical design loading conditions to assess are schematized in Figure 2b, where most of the imposed actions are expected to result in positive bending effects for the beams, while potential negative bending effects (i.e., due to suction wind) should also be taken into account. As long as the geometrical and mechanical features of the glass members and restraints in use are properly assessed, the LTB performance of LR systems according to Figure 2 is rationally expected to differ from a LU member with similar features. The LR features, moreover, can have substantially different efficiency, depending on the geometry of the members to verify.

Studies of the literature were reported in [27,28,29] for LR glass beams with continuous adhesive joints, giving evidence of the expected effects (and related calculation approaches) due to the presence of linear connections. In [29], the attention of finite element (FE) numerical analyses focused on LR glass beams with an adhesively restrained top edge, considering downward (i.e., positive bending moment effects) or upward (negative) design loads, as well as related LTB phenomena, in combination with various LR configurations. Luible and Schärer [30] also explored, via full-scale experiments and FE analyses, the LTB performance of glass beams (both monolithic and laminated) with discrete mechanical lateral restraints. The chosen geometrical configuration for the connectors in use (i.e., eight special round aluminum devices, glued to the edge of glass specimens), however, was associated with a mostly rigid and continuous LR effect. The actual contribution and the potential of local restraints for glass beams in LTB were also preliminary explored in [31].

In this paper, the elastic critical buckling moment of LR glass beams in LTB is investigated, with careful consideration of the effects of discrete mechanical restraints (i.e., Figure 2). As known, the knowledge of critical buckling loads for load-bearing systems generally represents poor information for accurate design purposes. Often, however, the availability of simplified (but accurate) calculation approaches can offer robust support to designers. Moreover, the accurate estimation of the expected critical buckling loads represents a key design step toward the definition (or calibration) of standardized calculation procedures and recommendations of practical use [25]. To this aim, extended parametric analytical and FE numerical calculations are presented in this paper, for a wide set of geometrical and mechanical configurations of technical interest. The major advantage is preliminary derived from analytical methods of the literature [32,33], as well as from the LTBeam software [34] (even specifically developed for LR steel members). The LTB performance of LR glass beams having different geometrical features and their sensitivity to variations in the number and position of discrete mechanical restraints are, thus, assessed. More extended parametric calculations are, thus, carried out with the support of ABAQUS software [35], so as to allow for a more refined and/or generalized description of the typical LR configurations in the field of structural glass applications.

To this aim, Section 2 firstly summarizes the current approach for the LTB design of LU glass members. In Section 3, the reference (steel-related) calculation methods are described. In Section 4, the attention is focused on the characterization of selected point fixings in use for glass applications, giving evidence of the expected stiffness parameters and the reliability of simplified, spring-based methods. Finally, Section 5 and Section 6 summarize the major outcomes of the extended parametric calculations for LR glass members.

## 2. LTB Design of Structural Glass Beams

### 2.1. General Approach for Laterally Unrestrained (LU) Beams

**Figure 3:**
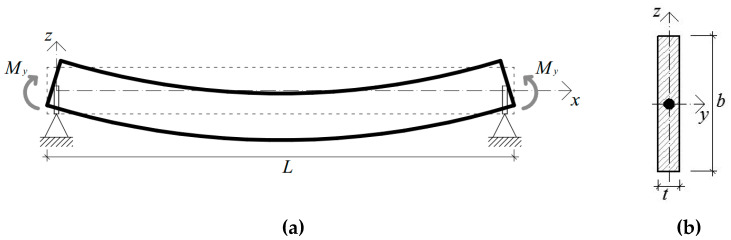
Lateral–torsional buckling (LTB) reference system for a laterally unrestrained (LU) monolithic beam under constant bending moment *M*_y_: (**a**) front view and (**b**) nominal cross-section.

For most existing design standards, guidelines, and regulations for structural beams in LTB, the reference calculation methodology assumes that the member to verify is laterally unrestrained (LU), with fork-torsional end supports (Figure 3). The possible effects due to adjacent or interconnected constructional members (i.e., roof panels, etc.) are, hence, fully disregarded, in favor of an LTB verification of independent members.

Based on literature studies and international design standards for steel structures [36], buckling failure phenomena of structural members according to Figure 3 can be conventionally prevented by means of standardized empirical verification methods, which, in most cases, are developed on standardized design buckling curves. The effective LTB design resistance *M*_b,Rd_ of the member in Figure 3 can be in fact estimated so as to satisfy the following limit condition:

(1) $$M_{b,\mathrm{Rd}}=\chi_{\mathrm{LT}}\frac{W_{y}f_{\mathrm{yk}}}{\gamma_{M1}}\geq M_{y,\mathrm{Ed}},$$

where *M*_y,Ed_ is the design bending moment, $W_{y}$ is the elastic resistant modulus for the *t* × *b* resisting cross-section, $f_{\mathrm{yk}}$ is the ultimate (tensile) resisting stress, and *γ*_M_ is a material partial safety factor.

A key input parameter in Equation (1) is represented by the buckling reduction coefficient $\chi_{\mathrm{LT}}$ ($\chi_{\mathrm{LT}}\leq 1$) and, thus, by the normalized slenderness ratio of the structural member to verify.

(2) $${\bar{\lambda }}_{\mathrm{LT}}=\sqrt{\frac{W_{y}f_{\mathrm{yk}}}{M_{\mathrm{cr}}^{\left(E\right)}}},$$

with

(3) $$M_{\mathrm{cr}}^{\left(E\right)}=M_{\mathrm{cr},0}=\frac{n\pi^{2}EI_{z}}{L_{}^{2}}\sqrt{\frac{I_{w}}{I_{z}}+\frac{L^{2}GI_{t}}{\pi^{2}EI_{z}}},$$

Euler’s theoretical buckling moment.

In Equation (3), *E* and *G* denote the Young’s and shear moduli, respectively, *I*_z_ signifies the moment of inertia about the minor *z*-axis, and *I*_t_ is the torsional moment of inertia for the monolithic *t* × *b* cross-section, with *I*_w_ being its warping stiffness (where *I*_w_→0 for rectangular cross-sections). At the same time, *n* = 1 is the integer value able to minimize—for a general set of geometrical and mechanical properties—the expected $M_{\mathrm{cr},0}$ value.

As long as Equations (2) and (3) are known, the required buckling reduction coefficient in Equation (1) is given by

(4) $$\chi_{\mathrm{LT}}=\frac{1}{\varphi_{\mathrm{LT}}+\sqrt{\varphi_{\mathrm{LT}}^{2}-{\bar{\lambda }}_{\mathrm{LT}}^{2}}}\leq 1.$$

The latter equation, as known, is sensitive to several aspects, such as ${\bar{\lambda }}_{\mathrm{LT}}$, as well as to the amplitude of initial geometrical imperfections, to the effects of possible residual stresses, and/or to load eccentricities. In Equation (4), all these initial defects are conventionally accounted for as

(5) $$\varphi_{\mathrm{LT}}=0.5\;\left[1+\alpha_{\mathrm{imp}}\left({\bar{\lambda }}_{\mathrm{LT}}-\alpha_{0}\right)+{\bar{\lambda }}_{\mathrm{LT}}^{2}\right],$$

where α_imp_ and α_0_ are appropriate imperfection factors (see [36] for the reference values in use for steel sections). 

**Figure 4:**
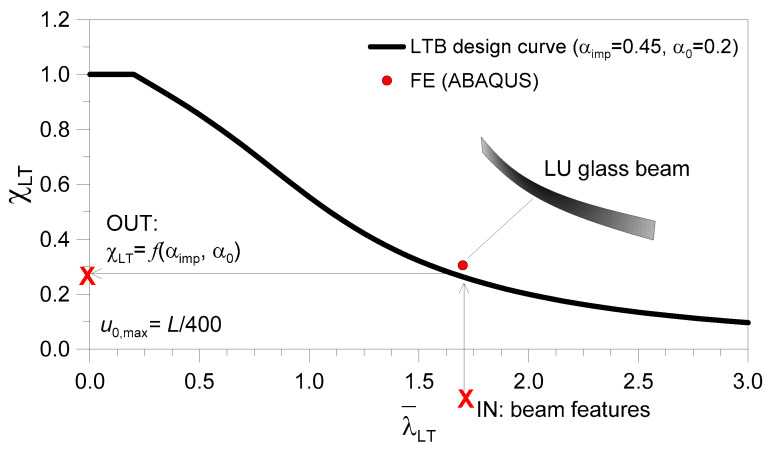
Standardized LTB design approach proposed in [25] for LU glass beams (with *u*_0,max_ = *L*/400, the maximum amplitude of the reference sine-shaped imperfection).

In similarity to LU steel members in LTB, design buckling curves were proposed in [25] for LU beams composed of glass. In particular, assuming the characteristic bending tensile resistance of glass *f*_yk_ = *σ*_Rk_ in (Equation (1)), with *γ*_M_ = 1.4, it was shown in [25] that reliable LTB calculations can be carried out as long as the above imperfection factors are set to α_imp_ = 0.45 and α_0_ = 0.20 (for equivalent initial sine-shaped imperfections of maximum amplitude, *u*_0,max_ = *L*/400). As long as the design constant bending moment *M*_y,Ed_ in Equation (1) is replaced by a mid-span concentrated load *F* or a uniformly distributed load *q*, the same design approach can still be adopted for glass members in LTB [25]. The standardized verification method is also implemented—with appropriate safety factors—in the set of design recommendations provided in [5] for glass structures. The basic assumption of the design approach is schematized in Figure 4.

Worthy of interest is that the above LTB verification approach can be practically extended to laminated glass sections, by accounting for the viscoelastic foils (under well-defined time/temperature conditions) via an equivalent secant shear modulus *G*_int_ [37,38,39]. A key step, however, is represented by the reliable calculation of the section properties (especially *I*_z_ and *I*_t_). Among others, the equivalent glass thickness *t*_eq_ can be derived from Table 1; Table 2 (with *t*_1_, *t*_2_ and *t*_int_ representing the thicknesses of glass and interlayers).

### 2.2. Laterally Restrained Beams with Continuous Adhesive Connections

**Figure 5:**
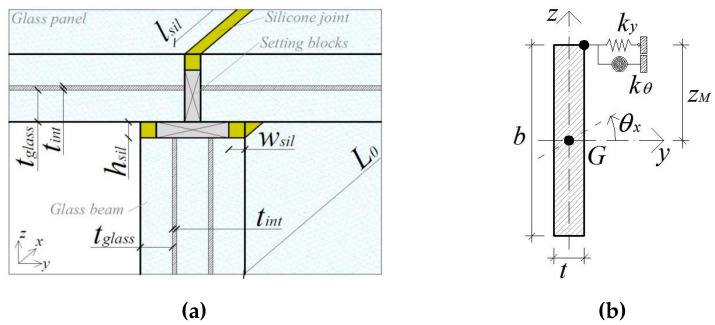
LR glass beams with continuous flexible lateral restraints: (**a**) example of a beam-to-plate adhesive joint, in accordance with [27], and (**b**) reference cross-section. Figures reproduced from [27] with permission from Elsevier, Copyright© license number 4813131416995, April 2020.

As long as the glass beam in Figure 3 is characterized by the presence of lateral restraints agreeing with Figure 5a, more accurate calculation approaches are required for reliable predictions. From a mechanical point of view, the resisting cross-section for the LR member in LTB is schematized in Figure 5b.

It was shown in [27,28,29] that adhesive joints in use for structural glass applications are typically associated with limited stiffness parameters. The presence of (even weak) continuous flexible joints according to Figure 5a, however, can have marked effects on the actual LTB response of a given glass beam. Compared to a basic LU member, the response of the LR element is in fact associated with a modified buckled shape, which can further modify the number of half sine-waves, as long as the adhesive linear restraints are progressively able to prevent possible lateral deformations [27,28,29].

In terms of LTB design procedure, the presence of the continuous adhesive restraint in Figure 5 manifests in the increase of the critical buckling moment $M_{\mathrm{cr},R}\geq M_{\mathrm{cr},0}$ (with “R” denoting the presence of lateral restraints), as well as in a modification of the fundamental buckling shape, compared to Figure 4. Given the shear stiffness contribution *k*_y_ of the linear restraint (with *k*_θ_ = 0), it was, thus, proven in [27] that $M_{\mathrm{cr},R}$ for a general cross-section can be reliably calculated as

(6) $$M_{\mathrm{cr},R}=z_{M}k_{y}{\left(\frac{L}{n_{R}\pi }\right)}^{2}+\sqrt{\left[EI_{z}{\left(\frac{n_{R}\pi }{L_{0}}\right)}^{2}+k_{y}{\left(\frac{L}{n_{R}\pi }\right)}^{2}\right]\left[EI_{w}{\left(\frac{n_{R}\pi }{L}\right)}^{2}+GI_{t}+z_{M}^{2}k_{y}{\left(\frac{L}{n_{R}\pi }\right)}^{2}\right]},$$

where *n*_R_ ≥ 1 is the integer number of half-sine waves able to minimize Equation (6), while *z*_M_ is the distance between the lateral restraint and the longitudinal axis of the beam (Figure 5b). 

It is important to note that, as long as *M*_cr,R_ is modified in Equation (6), the normalized slenderness ratio ${\bar{\lambda }}_{\mathrm{LT}}$ in Equation (2) is also modified. Accordingly, the susceptibility of a given LR glass member to LTB phenomena (i.e., Equation (4)) can be reasonably different from that of an identical LU geometry. In any case, it was shown in [27] that Equations (1)–(5) and Figure 4 can be still adopted for LTB design purposes, as long as the actual *M*_cr,R_ value is properly estimated (Equation (6)) and possible global imperfections for the element to verify do not exceed the reference equivalent amplitude (*u*_0,max_ = *L*_0_/400).

### 2.3. Laterally Restrained Beams with Discrete Mechanical Connections

**Figure 6:**
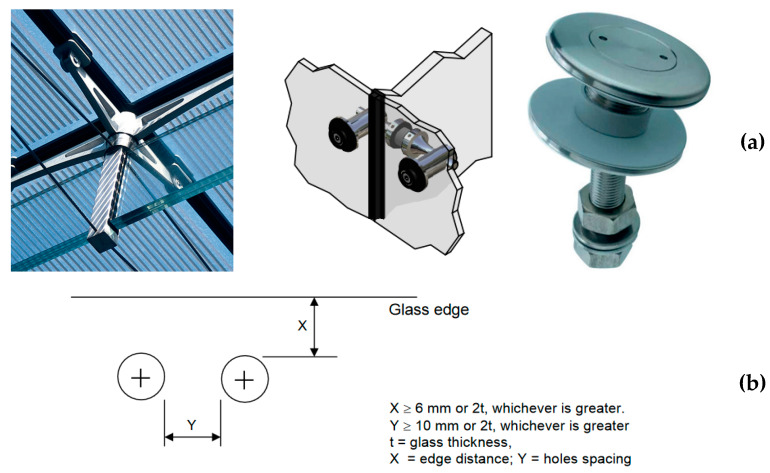
LR glass beams with discrete mechanical lateral restraints: (**a**) examples of point fixings and (**b**) schematic representation of minimum distance requirements for the glass holes.

When discrete mechanical restraints according to Figure 2 or Figure 6 are used for glass beams, further design issues must be unavoidably assessed, compared to Section 2.1 and Section 2.2.

The actual role and LTB efficiency of discrete restraints agreeing with Figure 6a (that could be variably spaced and positioned along the span of a glass member) should in fact be properly characterized. Under ordinary design loads, moreover, major calculation troubles for resistance verifications may usually derive from the presence of glass holes, which should respect appropriate geometrical prescriptions (Figure 6b). In the multitude of mechanical devices that are commercially available for structural glass applications, the goal of design practice is, in fact, to avoid premature cracks in glass, as well as to facilitate the installation stage. Stiff metal connectors agreeing with Figure 6a, as such, generally use soft layers and gaskets that are interposed to the edges of glass, in order to accommodate local relative displacements and to protect the edges from potential stress peaks. From an LTB calculation perspective, all the above aspects manifest in the form of additional local stiffness (of devices) and flexibility (due to gaps/gaskets) that should be properly taken into account, even for the prediction of the theoretical critical buckling moment $M_{\mathrm{cr},R}$.

## 3. LTB Theoretical Background and Solving Methods for LR beams with Discrete Mechanical Restraints

In the last few decades, several research projects focused on the LTB analysis of (non-glass) load-bearing members with discrete mechanical restraints. Most efforts were specifically aimed at steel load-bearing systems, offering accurate theoretical formulations and simplified analytical approaches in support of designers [32,33,40,41,42]. A brief overview of suitable methods is proposed in this section.

Among the available calculation tools that could be extended to LR glass members in LTB, the first issue is related to the accurate prediction of the theoretical critical moment *M*_cr,R_. In addition to the basic variations in the intrinsic material properties, compared to steel girders, glass beams are in fact expected to have relatively higher length-to-thickness and length-to-height ratios, thus further enforcing their susceptibility to possible LTB phenomena. Further key parameters to account for in LTB calculations of LR glass members are then represented by the following mechanical restraints in use (*rotules*, etc.).

- their number (generally *n*_b_ > 1),
- the spacing *s* and position (with the respect of minimum edge-distance of glass holes, etc.),
- the detailing features, for devices that are specifically designed for glass applications (i.e., Figure 6),
- finally, the presence of gaps and soft gaskets at the glass-to-restraint interface, as typically in use to prevent local stress peaks in the region of holes.

### 3.1. Reference Theoretical Formulation for I-section Steel Beams

**Figure 7:**
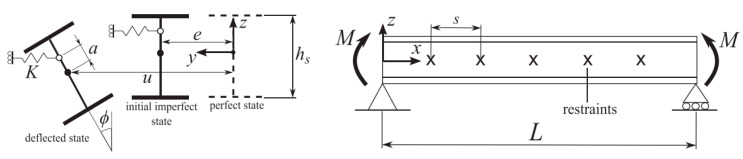
Reference analytical model for LR beams with discrete rigid lateral restraints. Figure adapted from [33] with permission from Elsevier, copyright© license number 4813141188013, April 2020.

Extended studies for the LTB analysis of steel beams with discrete restraints were carried out in [32,33]. Following Figure 7, the critical buckling moment of a simply supported, doubly symmetric I-section steel beam with span *L* and a number *n*_b_ of discrete, elastic lateral restraints (with infinitely rigid stiffness *K*) is generally recognized to be

(7) $$M_{\mathrm{cr},R}^{}=M_{T}=\frac{\pi^{2}EI_{z}}{s^{2}}\sqrt{\frac{I_{w}}{I_{z}}+\frac{s^{2}GI_{t}}{\pi^{2}EI_{z}}},$$

where Equation (8),

(8) $$s=\frac{L}{n_{b}+1},$$

denotes the uniform distance of the *n*_b_ discrete restraints; thus, Equation (9),

(9) $$M_{\mathrm{cr},R}^{}=\left(n_{b}+1\right)\cdot M_{\mathrm{cr},0},$$

is the upper theoretical limit for the expected buckling resistance, compared to Equation (3).

The above formulation assumes that the so-called “threshold” moment *M*_T_ represents the bending moment for which the LR beam buckles in (*n*_b_ + 1) half-sine waves. In other words, the stiffness *K* of *n*_b_ ≥ 1 restraints is so high that a fully rigid lateral bracing can be offered to the beam to verify, i.e., with null lateral displacements at the locally restrained nodes. As such, the same *M*_T_ value is equivalent to the critical moment of an LU beam segment with identical cross-section properties but total span *s* (see Equation (8)).

Major issues for the LTB analysis of a general steel girder (see [32,33]) are thus represented by the “rigidity efficiency” of the discrete restraints in use and, thus, by the detection of possible intermediate configurations in which certain nodal displacements could be expected in the region of discrete restraints. McCann et al. [33], for example, solved a linear Rayleigh–Ritz analysis and proposed an extended formulation for the LTB analysis of general LR steel beams. According to [33], it was shown that, once *K* exceeds the “threshold” stiffness value *K*_T_, the lateral restraints in use have a full bracing effect; thus, Equation (10),

(10) $$K\geq K_{T},$$

reflects a critical buckling moment $M_{\mathrm{cr},R}$ agreeing with Equation (7). To this aim, the threshold stiffness value must be estimated as

(11) $$K_{T}=\left(\frac{EI_{z}}{s^{3}}\right)\frac{62\;\left(1+\kappa_{s}\right)}{A_{0}+A_{1}\overset{◠}{a}},$$

with the equations below denoting the normalized distance between the discrete restraints and the section shear center (with *h*_s_ representing the total height of the I-shaped profile in Figure 7).

(12) $$\kappa_{s}=\frac{\kappa }{{\left(n_{b}+1\right)}^{2}},$$

(13) $$\kappa =\frac{L\;GI_{t}}{\pi^{2}EI_{w}},$$

(14) $$a^{*}=\frac{2a}{h_{s}},$$

Finally, according to [33],

(15) $$A_{0}=0.45+2.8\;\nu_{b,T}\;\kappa_{s},$$

(16) $$A_{1}=6.3\;\nu_{b,T}+2.2\;\kappa_{s}-1,$$

(17) $$\nu_{b,T}=\frac{1}{1+cos\left[\left(\pi /n_{b}+1\right)\right]}.$$

More in detail, *v*_b,T_ in Equation (17) is respectively equal to 1.0 for *n*_b_ = 1, 0.667 for *n*_b_ = 2, and 0.586 for *n*_b_ = 3, while it tends progressively to the lower limit *v*_b,T_ = 0.5 for higher *n*_b_ values.

Following Figure 7, a key condition for Equations (7)–(17) is that the discrete restraints in use are positioned at a height *a* above the shear center of the cross-section object of analysis (with positive distance values for bracing systems on the compression side), such that the following equation applies [33]:

(18) $$a\geq a_{\lim}=\frac{h_{s}+\kappa_{s}}{4\sqrt{1+\kappa_{s}}}.$$

### 3.2. Linear Interpolation Approaches

According to Equation (7), it is rationally expected that the critical buckling moment of a given LR member in LTB can increase significantly, as long as rigid lateral restraints (with *n*_b_ ≥ 1 and *s* < *L*) are able to prevent possible displacements of the involved nodes. At the same time (see Equations (7) and (9)), $M_{\mathrm{cr},R}$.progressively decreases for *K* < *K*_T_, and finally returns to the limit condition represented by the LTB response of a simple LU beam (see Equation (3)).

Several research studies were carried out over the years for the LTB assessment of LR steel girders, where relatively stiff purlins of other bracing systems were used to provide stabilization. Major calculation troubles arise when node-displacing critical modes can occur, since closed-form solutions cannot be analytically derived for the reliable prediction of the critical moment $M_{\mathrm{cr},R}$. McCann et al. [33], in this context, proposed a conservative, linear approximation for intermediate stiffnesses (i.e., 0 < *K* < *K*_T_), so that the corresponding *M*_cr,R_ value could be reliably estimated as

(19) $$M_{\mathrm{cr},R}=M_{\mathrm{cr},0}+\frac{K}{K_{T}}\left(M_{T}-M_{\mathrm{cr},0}\right),$$

with *M*_cr,0_ and *M*_T_ representing the unrestrained and threshold critical values given by Equations (3) and (7), respectively. Past comparative calculations and validation studies discussed in [32,33] proved that Equation (19) can be suitable for the LTB design of general steel beams. However, the same studies highlighted that a higher degree of accuracy can be expected, especially for steel members with a braced compression flange and with a single restraint (*n*_b_ = 1) at the mid-span section.

An alternative linear fitting approach was suggested by Trahair [43], for LR beams with a single restraint (*n*_b_ = 1). The latter, however, can be applied with accuracy to beams with a discrete restraint on the shear center (i.e., $a^{*}=0$ in Equation (14)). Many other approximation proposals are also available in the literature, and they are specifically validated for a given number, position, and stiffness of discrete restraints in steel members. Parametric studies were discussed in [42], for steel members under various loading and LR conditions of technical interest for design. Extended parametric analyses were presented in [29] for steel beams under various LR configurations, but giving evidence of the intrinsic advantages or limits for several linear interpolation approaches of the literature. 

In this research paper, parametric calculations are, thus, proposed for several LR glass members in LTB. As a reference toward more refined calculations, the linear interpolation approach in Equation (19) is taken into account. 

### 3.3. LTBeam Tool for Steel Beams

The LTBeam tool is firstly considered as an alternative calculation method. The software, more in detail, was developed at CTICM (Centre Technique Industriel de la Construction Metallique, *www.cticm.com* [34]), as part of a past European research project. The original goal of this tool is to compute the elastic critical moment *M*_cr,R_ of LR steel beams with general cross-sectional features, in any number of different load cases and boundaries, according to the finite element (FE) method.

For a given cross-section (both mono- or double-symmetric), the assigned beam to verify is discretized into a maximum of 300 FE elements. Non-steel members can also be efficiently investigated, once the flexural, torsional, and warping cross-section properties are defined in the input parameters, together with the Young’s modulus and Poisson’s ratio for the material in use. The presence of LR mechanical restraints (up to *n*_b_ = 2), finally, can be described in the form of equivalent elastic springs with input stiffness and position. When alternative calculation methods are not available, an intrinsic advantage of the LTBeam tool is that the expected *K*_T_ value in Equation (19) can be numerically derived by iteration, i.e., as the minimum stiffness *K* that leads *M*_cr,R_ to coincide with the threshold critical moment *M*_T_ for the member object of analysis.

### 3.4. General FE Numerical Method

When the input geometrical details for the system to verify do not match with the approaches summarized in Section 3.2 and Section 3.3, commercially available computer software codes can be adapted to specific configurations.

**Figure 8:**
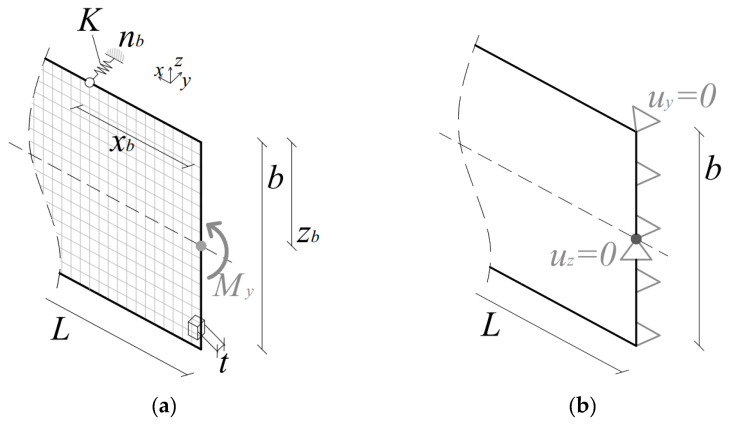
Preliminary finite element (FE) numerical model for linear bifurcation analyses on LR glass beams in LTB (ABAQUS).

In this paper, for example, a set of parametric linear buckling analyses were developed with the ABAQUS/Standard software (“buckle–linear perturbation” step). Through the parametric investigation, the same modeling technique was taken into account to describe various LTB configurations characterized by different number, position, and stiffness of discrete restraints, among a wide series of beam geometries. The typical FE model consisted of S4R four-node, quadrilateral, stress/displacement shell elements with reduced integration and large-strain formulation (S4R type of ABAQUS element library; see Figure 8a). A regular mesh pattern was used, with *l*_mesh_, the characteristic size of quadrilateral elements, comprised between 2 mm and 5 mm, depending on the global dimensions *b* × *L* of the studied beams. Given the goal of linear bifurcation analyses, glass was described as an isotropic, indefinitely linear elastic material. 

Boundary conditions for the simply supported, fork-end supported beams in LTB were described via equivalent nodal constraints, so as to reproduce the reference set-up in Figure 3. Restraints were used for both the shear center of the beam end sections (*u*_y_ = 0, *u*_z_ = 0),and all the end section nodes (*u*_y_ = 0), as shown in Figure 8b. Accordingly, bending moments *M*_y_ were introduced at the barycentrical node of the end sections in Figure 8. A “*coupling*” kinematic constraint was used to redistribute the so-defined bending moments on the section height *b*.

According to several literature contributions on LR steel members in LTB, the bracing effect of discrete mechanical restraints was numerically reproduced in a simplified way, i.e., by means of a number *n*_b_ of linear elastic springs (“*axial*” type) with stiffness *K*. These springs were introduced to brace the compression edge of the examined glass beams, at a given distance *z*_b_ from the shear center. Single restraints (*n*_b_ = 1) were placed at the mid-span section of the selected geometries (*x*_b_ = *L*/2). In presence of multiple restraints (*n*_b_ > 1), an equal spacing *s* was taken into account (Equation (8)). The detailing of holes in glass, finally, was disregarded for the elastic LTB analysis of the so-assembled spring-based models.

## 4. Mechanical Characterization of LR Glass Beams with Discrete Restraints

### 4.1. Design Issues

When the calculation approaches summarized in Section 3 are taken into account for LTB calculations, an open issue for glass members is certainly related to the actual effects of real mechanical restraints. According to Figure 6, most of them are in fact characterized by geometrical and mechanical features that often hardly match with idealized nodal elastic springs.

**Figure 9:**
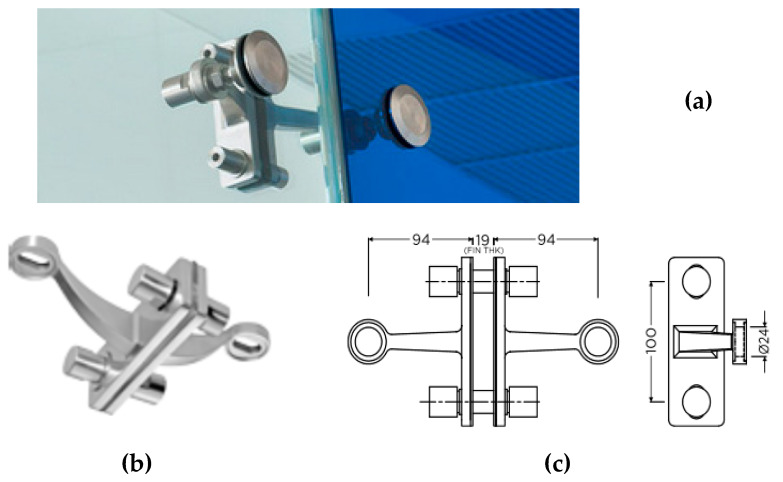
Example of two-way spider mechanical restraint for glass members: (**a**) global assembly with rotules; (**b**) axonometry detail; (**c**) nominal dimensions (top and side views, values in mm).

As such, a preliminary attempt of this project was, thus, dedicated to the assessment of nominal features for some of the discrete restraints that are actually used for structural glass fins in roofs and facades. In addition to the multitude of commercially available solutions, the attention was focused on the selected configuration in Figure 9. The latter includes a stainless-steel device (AISI 316 alloy type) and is intended for glass fins up to 19 mm in total thickness. Two 100-mm-spaced holes are used to fix the steel plates to the glass section. Two lateral rigid bars for the spider device allow then the connection between the glass fin and the orthogonal plates (point fixing *rotules*). To avoid local stress peaks in glass, Teflon® (polytetrafluoroethylene, PTFE) washers are interposed onto the glass beam and the metal components. Such a design solution is in line with the point fixing practice of glass structures, where various low-modulus soft layers can be used. Possible alternatives involve nylon, polyoxymethylene (POM), ethylene propylene diene monomer (EPDM), or other rubber materials [1]. Dedicated studies, accordingly, should be generally spent for the characterization of different restraint solutions. Usually, the above details result from accurate design and resistance verification steps [1]. Given that the low-modulus materials in use can be sensitive to time loading and temperature, their mechanical properties should be properly assessed, with the support of literature documents or experiments [44,45,46,47,48,49]. For preliminary LTB estimates, however, these soft layer properties can be assumed as equivalent linear elastic terms.

### 4.2. FE Buckling Analysis and Stiffness Estimate for LR Glass Members

**Figure 10:**
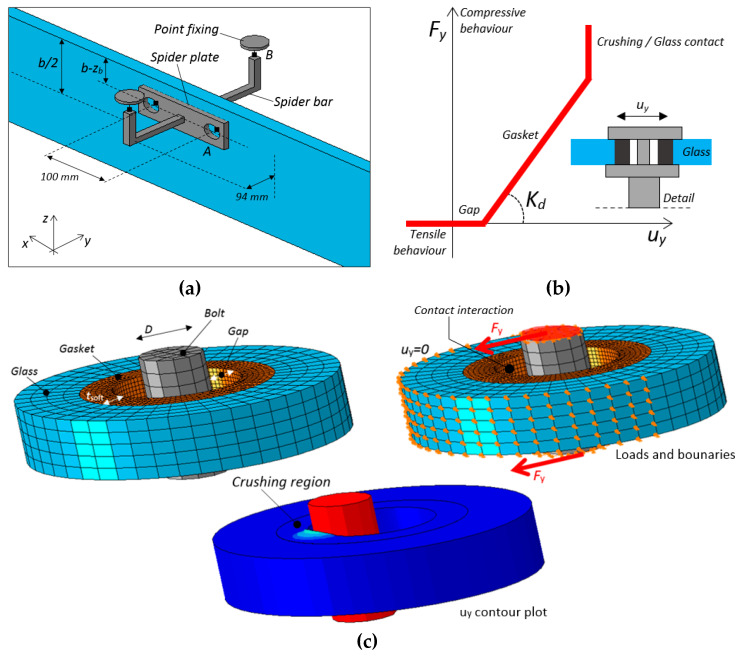
FE numerical modeling of glass beam in LTB (*n*_b_ = 1, *x*_b_ = *L*/2): (**a**) restraint detail, with evidence of (**b**) mechanical characterization of gaskets (detail “B”), and with (**c**) example of local FE model for the stiffness analysis of a “detail B” point-fixing (ABAQUS).

To solve the above issue and assess the reliability of simplified spring-based FE calculations, the modeling approach described in Section 3.4 was, thus, further elaborated to include a more refined description of restraint details. The example in Figure 10, in particular, was developed by taking inspiration from Figure 9. From a mechanical point of view, the latter represents a single discrete restraint (*n*_b_ = 1) composed of two point fixings (*n*_d_ = 2) with a certain local stiffness *K*_d_.

For the FE analysis, a set of solid brick elements were, thus, used to reproduce the device components. The materials in use (glass and steel) were described via linear elastic constitutive laws, with *E* = 70 GPa, *ν* = 0.23 for glass [50] and *E*_s_ = 197 GPa, *ν*_s_ = 0.3 for steel [51]. Two spider plates similar to Figure 9 were firstly introduced in the FE assembly (see Figure 10a). The 100-mm-spaced bolts were reproduced via kinematic constraints (“beam MPC” option from the ABAQUS library) able to offer a rigid axial and rotational restraint to the involved opposite faces, in the region of holes (see the detail “A” in Figure 10a). At the same time, a frictionless surface contact interaction was interposed at the adjacent glass-to-steel surfaces (i.e., where soft, frictionless gasket layers are used). 

The lateral rigid bars of the spider device in Figure 9, finally, were schematized in the form of squared-shape geometries. Compared to the nominal device in Figure 9, an average cross-sectional area was taken into account, given that the length and height of these bars was respected. Above these bars (see the detail “B” in Figure 10a), two point fixings were in fact introduced in the form of solid discs. For each one of them, the middle axis was placed at a distance of 94 mm from the external face of the beam. The steel plates and the corresponding lateral bars were ideally “tied”, thus enabling possible relative deformations.

Special care was dedicated to the description of the mechanical interaction between the spacer bars and the above point fixings. To this aim, a combined “*axial + rotational*” connector was introduced between each spider bar and the corresponding top plate (detail “B” of Figure 10a). Such an assumption was chosen to characterize the nominal geometrical detail in Figure 10b, where the device and the glass hole (of the braced orthogonal glass plate) must interact via the interposed gap and soft gasket. The schematic drawing in Figure 10b, in this regard, adequately reproduces the theoretical behavior for the examined point fixing detail and a given glass beam in LTB. The connector, as usual, does not react in tension. Otherwise, on the compression side, it is expected to progressively enable possible lateral displacements of the beam. At first, the assigned gap allows a certain accommodation of local deformations (*K*_d_ = 0). When the contact initiates between the bolt device and the adjacent soft layer, a certain stiffness *K*_d_ is indeed offered against possible lateral deformations of the beam. This local stiffness contribution, however, could be susceptible to local crushing of the soft layer, thus requiring specific design of geometrical details. 

The reliable calculation of *M*_cr,R_ for a given LR member, according to Section 3, requires that the global elastic stiffness *K* for the spider device should be estimated first.

For practical LTB calculations, it is assumed that each detail agreeing with Figure 10a,b (where *n*_b_ = 1 and *n*_d_ = 2) is the result of multiple local stiffness contributions *K*_d_ (if *n*_d_ > 1) that can be placed at different *x*_b_ positions of the span *L* for the LR beam to verify. The cumulative stiffness *K* due to the mechanical restraint in Figure 10 is, thus, expected to be

(20) $$K=\overset{n_{d}}{\underset{d=1}{\sum }}K_{d},$$

where Equation (21),

(21) $$K_{d}=\frac{F_{y}}{u_{y}},$$

represents the equivalent stiffness for a single point fixing. The latter depends on the Young’s modulus of the soft layer in use (*E*_soft_), its thickness *t*_soft_, and the contact surface in compression (*A*_soft_), in the region of interaction between the bolt and the glass hole. Accordingly, dedicated experimental analyses or refined FE calculations should support the accurate characterization of local mechanical behaviors (Figure 10c). As long as the calculations are limited to the linear elastic regime, however, an approximate prediction of this local stiffness *K*_d_ can be obtained as

(22) $$K_{d}\approx \frac{E_{\mathrm{soft}}\;A_{\mathrm{soft}}}{t_{\mathrm{soft}}},$$

with

(23) $$\;A_{\mathrm{soft}}\approx t_{\mathrm{soft}}\;0.8D,$$

and *D* the nominal diameter of the bolt.

It is, thus, clear that, as long as the input parameters in Equations (22) and (23) are modified, a markedly different prediction can be achieved for the total stiffness *K* in Equation (20). The latter, in addition, can have different bracing effects and efficiency, depending on the geometry of the LR beam to verify.

**Figure 11:**
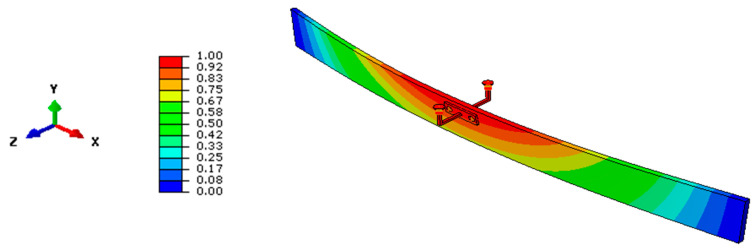
Example of fundamental critical shape (ABAQUS) for a glass beam in LTB (*n*_b_ = 1, *x*_b_ = *L*/2) with LR details according to Figure 10.

The example in Figure 11, in this regard, shows the typical deformed shape for a selected geometry (*t* = 20 mm, *b* = 200 mm, *L* = 2000 mm) and a single LR device according to Figure 10a (*n*_b_ = 1, *n*_d_ = 2, *x*_b_ = *L*/2, *z*_b_ = 55 mm). The deformed shape in Figure 11 specifically refers to a total elastic stiffness *K* = 40 kN/m (with *K*_T_ = 144 kN/m, *M*_cr,0_ = 18.69 kNm and *M*_T_ = 37.38 kNm).

From the deformed shape in Figure 11 (weak restraint), it is possible to notice that the spider device does not affect the global LTB response of the glass beam. In other words, as long as the total stiffness *K* is limited, compared to *K*_T_, local deformations are still allowed for the restrained nodes. From Table 3, moreover, it is possible to see the variation of critical moment estimates for the same beam geometry, as long as *K* increases. The collected values are obtained from the linearized analytical approach, the LTBeam tool, and the refined FE model in Figure 11.

As long as *K* increases, the bracing effect of local restraints manifests in a progressive increase of *M*_cr,R_, within the limit values *M*_cr,0_ and *M*_T_. The comparison of analytical, LTBeam, and more refined FE calculations in ABAQUS shows that the first approach tends to underestimate the expected bracing effect, while the general trend of numerical calculations is mostly in good correlation. For the selected example, the spring-based estimates show less than a 2% scatter from the more accurate FE model with three-dimensional (3D) restraints. 

A relevant calculation issue is, thus, represented by the threshold stiffness value *K*_T_ described in Equation (19), given that Equations (11)–(17) were specifically proposed in [32,33] for doubly symmetric I-section steel members. The reference analytical approach can be further adapted to rectangular *t* × *b* glass sections, as long as it is assumed that the warping stiffness in Equation (13) reduces to

(24) $$I_{w}\approx I_{w}^{*}=\frac{I_{z}\left(b^{2}\right)}{4}=\frac{b^{3}t^{3}}{48}.$$

Consequently (with *G*≈ *E*/3 the shear modulus of glass), the original Equation (13) can be expressed as

(25) $$k\approx \frac{16L}{\pi^{2}b^{2}}.$$

Once *k* is estimated from Equation (25), the final *K*_T_ value can, thus, be calculated via Equation (11). Compared to the exact formulation (i.e., Equation (13) or iterative calculations with the LTBeam tool, see Section 3.3), it is important to note that Equation (25) can result in rough threshold stiffness predictions for a given LR glass member. Therefore, careful consideration should be spent, at the design stage, to account for these intrinsic approximations.

**Figure 12:**
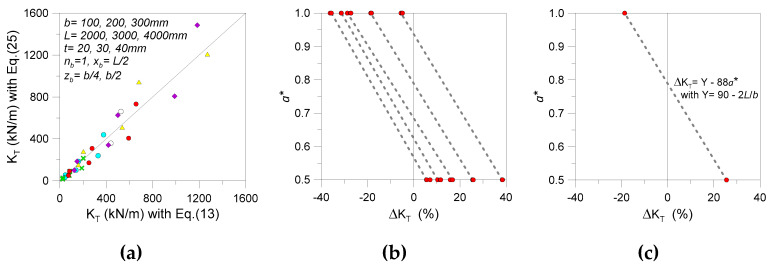
Comparative calculation of threshold stiffness *K*_T_ for LR glass beams, with evidence of (**a**) expected accuracy, (**b**) corresponding percentage scatter Δ*K*_T_ deriving from the adapted calculation approach (Equation (25), with selected results grouped by *L*/*b* and a*), and (**c**) correction proposal.

In this regard, comparative calculation examples are proposed in Figure 12a for selected LR configurations, while the corresponding percentage scatter Δ*K*_T_ (as obtained from Equation (25) or more accurate predictions) is shown in Figure 12b. The latter, given the assumption of Equation (25), can result in both positive or negative scatter (depending on the distance a^*^), and even in severe scatter. The combination of geometrical properties for the beam to verify, as well as the position of restraints, can both contribute to affect the final *K*_T_ result. According to Figure 12c, however, it is possible to see that the approximation in *K*_T_ calculated from Equation (25) can be efficiently addressed (for various glass beam geometries and restraint positions), as long as the suggested interpolation function is taken into account.

## 5. Analytical and FE Numerical Parametric Investigation

In accordance with Section 3 and Section 4, a series of parametric calculations were carried out on a wide set of geometrical and mechanical configurations. The critical buckling moment *M*_cr,R_ of monolithic glass beams subjected to a constant, positive bending moment *M*_y_ agreeing with the set-up in Figure 3 was predicted. Key input variations for the parametric study included modifications of the following:

- span *L* for the selected glass beams (with *L* = 2000, 3000, 4000 mm),
- cross-sectional dimensions *t* × *b* (with *t* = 20, 30, 40 mm and *b* = 100, 200, 300 mm),
- position *z*_b_ of restraints (with *z*_b_ = 0, *b*/4, or *b*/2),
- stiffness *K* of restraints (*K* = var).

Through the calculation steps, moreover, three different methods were assessed:

- simplified analytical calculations given by the modified linearized approach (i.e., Equation (19), with *K*_T_ from Equation (11) and *k* given by Equation (25) and Figure 12c).
- LTBeam estimates: for each glass member, the equivalent section properties and LR features were considered for the software input,
- FE (ABAQUS) models: based on Section 3.4 and Figure 8, where spring-based, *axial* connectors were used for the description of lateral restraints.

For all the above methods, the Young’s modulus of glass was set to *E* = 70 GPa, with *ν* = 0.23 as the Poisson’s ratio [50].

### Stiffness K and Position z_b_ of Single Discrete Restraints (n_b_ = 1)

**Figure 13:**
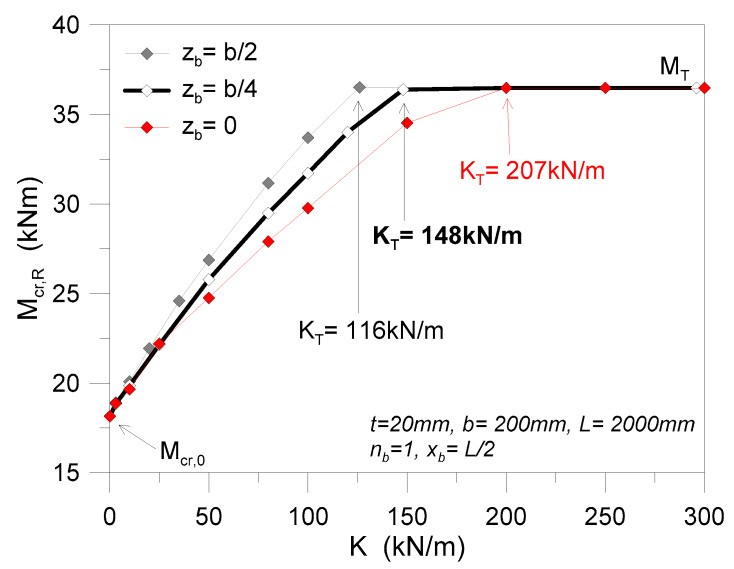
Comparative LTB calculations for selected LR glass beams with *n*_b_ = 1 discrete lateral restraints at different positions *z*_b_ (*K* = var), using ABAQUS.

A first series of comparative calculations was carried out for glass beams characterized by the presence of a single discrete restraint. Figure 13 shows the typical variation of critical moment *M*_cr,R_ for a given beam geometry (*t* = 20 mm, *b* = 200 mm, *L* = 2000 mm), as long as *K* is modified. 

**Figure 14:**
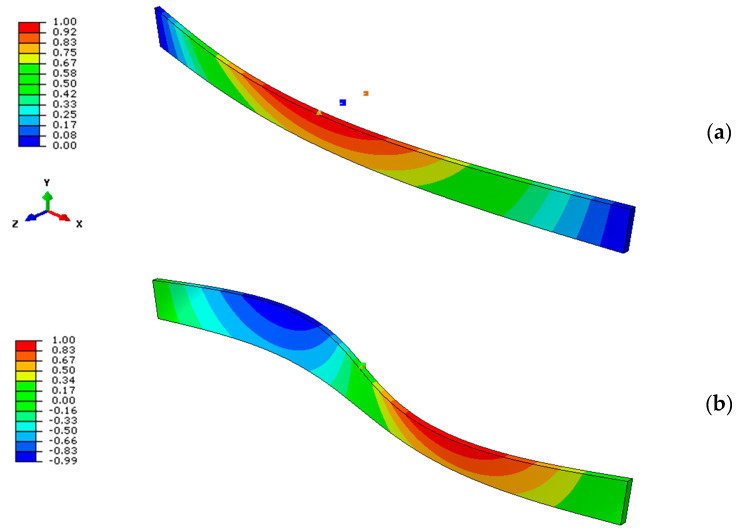
Typical LTB deformed shape for a selected LR glass beam (*L* = 2000 mm, *b* = 200 mm, *t* = 20 mm) with discrete lateral restraints (*n*_b_ = 1, with *x*_b_ = *L*/2 and *z*_b_ = *b*/2): (**a**) example of LU beam (*K*→0) and (**b**) LR limit condition (*K*→*K*_T_), using ABAQUS.

Together with the critical buckling moment, as expected, the deformed shape of the beam is progressively modified with *K*, from a single half-sine wave (weak lateral restraint, *K*→0) toward a double half-sine wave shape that is characterized by null displacements of locally restrained nodes (*K*→*K*_T_), as shown in Figure 14. Given that both the geometrical properties of the beam’s object of analysis and the restraint features can affect the final LTB predictions, additional calculations were, thus, carried out with the three selected methods earlier defined.

**Figure 15:**
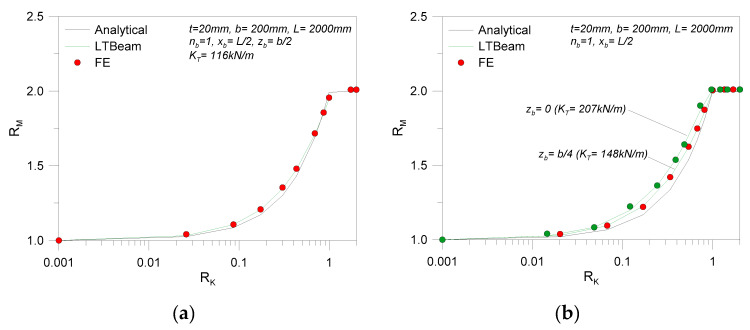
Comparative LTB calculations for LR glass beams with discrete lateral restraints (*n*_b_ = 1) at different positions *z_b_* (*K* = var): (**a**) *z*_b_ = *b*/2 and (**b**) *z*_b_ = 0, *b*/4.

Typical results are reported in Figure 15, in non-dimensional form, where the parameters in Equations (26) and (27),

(26) $$R_{M}=\frac{M_{\mathrm{cr},R}\;}{M_{\mathrm{cr},0}},$$

(27) $$R_{K}=\frac{K\;}{K_{T}},$$

denote the theoretical resistance increase and the stiffness efficiency for the discrete restraints in use, respectively.

**Figure 16:**
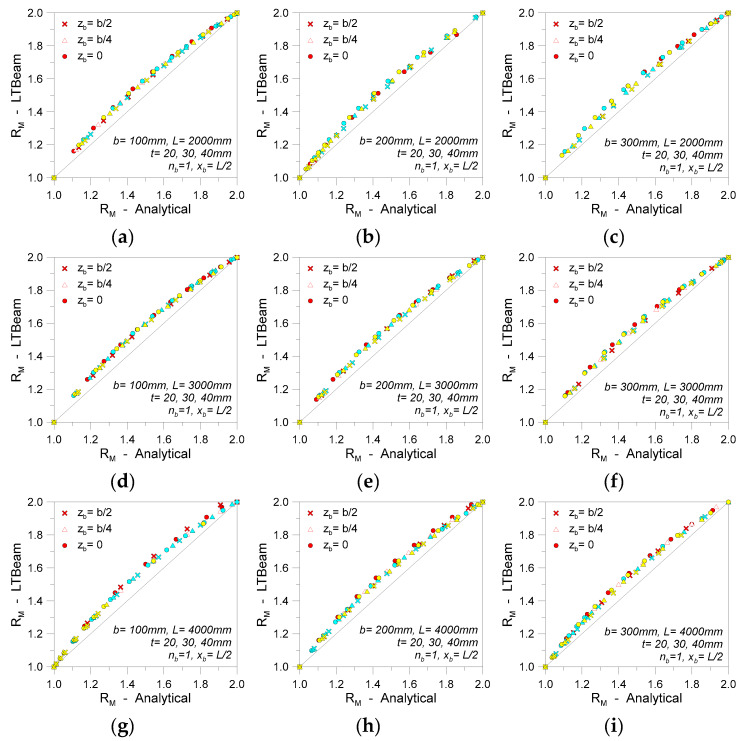
Comparative *R*_M_ calculations (Equation (26)) for LR glass beams in LTB (*n*_b_ = 1, *K* = var).

Figure 16, finally, presents the major outcomes of an extended analysis carried out on glass beams in LTB with a single discrete restraint (0 ≤ *K* ≤ *K*_T_). The collected results are compared in the form of *R*_M_ values (Equation (26)), as obtained for beam geometries grouped by span (*L* = 2000, 3000, 4000 mm), height (*b* = 100, 200, 300 mm), and thickness (*t* = 20, 30, 40 mm). Possible variations in the position of single restraints from the beam shear center are also taken into account (*z*_b_ = 0, *b*/4, or *b*/2, with *x*_b_ = *L*/2). When both the beam geometry and the restraint stiffness *K* are modified, it is interesting to note in Figure 16 a relatively stable trend for the *R*_M_ estimates from the numerical software or the “modified” linearized analytical approach.

For a more accurate quantification of the expected percentage scatter due to the intrinsic approximation from the analytical calculations, however, the percentage value in Equation (28) can be taken into account

(28) $$\Delta M_{\mathrm{cr},R}=100\cdot \frac{x_{\mathrm{num}}-x_{\mathrm{an}}\;}{x_{\mathrm{an}}}.$$

**Figure 17:**
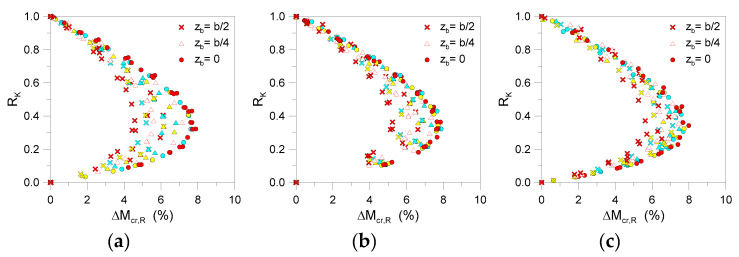
Percentage scatter (Equation (28)) for critical buckling moment estimates of selected LR glass beams (*n*_b_ = 1, *K* = var) grouped by span: (**a**) *L* = 2000 mm, (**b**) *L* = 3000 mm, and (**c**) *L* = 4000 mm (with *t* = 20, 30, 40 mm and *b* = 100, 200, 300 mm).

According to Figure 17, it is thus possible to note that the linearized analytical predictions are in rather good correlation with more detailed FE methods, for relatively stiff restraints (*K* > 0.8*K*_T_, for the examined systems).

The theoretical buckling moment of the selected LR beams is always underestimated by the linearized approach, as also expected. However, the percentage scatter from Equation (28) is less than 2% for most of the examined configurations. For intermediate *K* values (see Figure 17), the underestimation of LR bracing effects is maximized in the range of *K* = 0.3–0.4*K*_T_, with up to 7–8% scatter. As a result, the use of a linear approximated formulation is proven to offer reliable (and not severely conservative) predictions for the critical buckling moments for LR glass beams with single discrete restraints.

## 6. Analysis of Glass Beams with Multiple Discrete Restraints

At a subsequent stage, the parametric analysis was extended to LR glass beams characterized by the presence of multiple discrete restraints. Major results are grouped in Section 6.1 and Section 6.2, as a function of *n*_b_ (with 2 ≤ *n*_b_ ≤ 5).

### 6.1. Two Restraints (n_b_ = 2)

**Figure 18:**
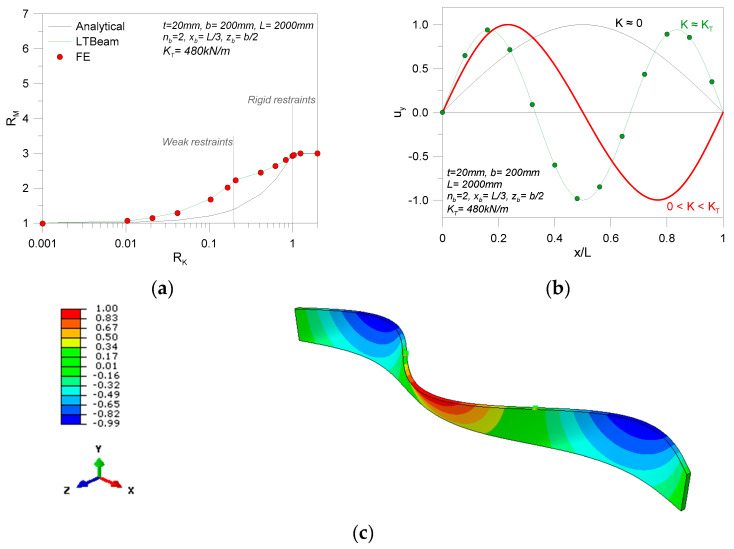
Comparative LTB calculations for LR glass beams with *n*_b_ = 2 discrete lateral restraints (with *K* = var): effects on (**a**) theoretical buckling resistance and critical deformed shapes (from (**b**) LTBeam or (**c**) ABAQUS respectively).

For glass beams with two equally spaced discrete restraints, most of the FE results and analytical calculations were proven to agree with the examples in Figure 18a. Therefore, it is possible to note that the linearized approach has a more pronounced underestimation of the corresponding FE estimates.

**Figure 19:**
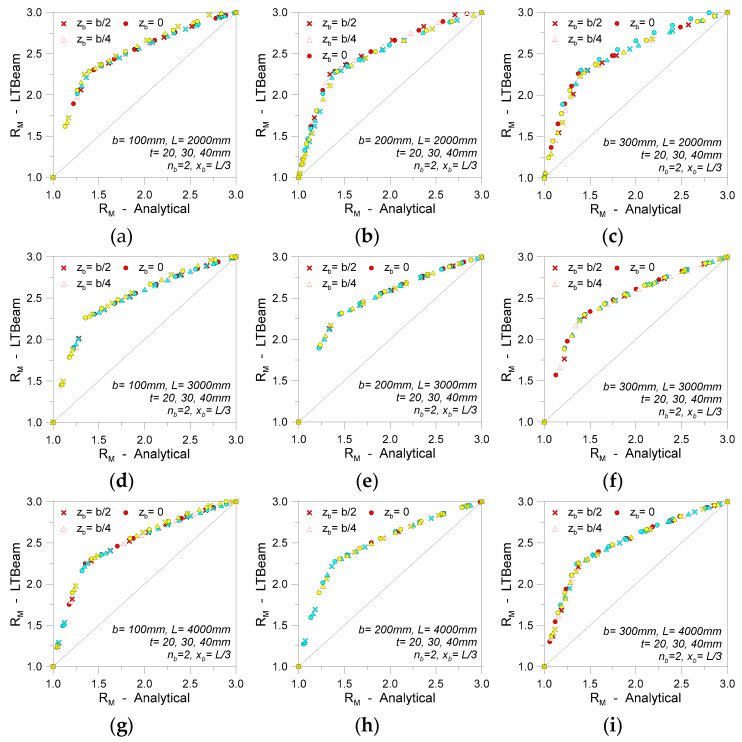
Comparative *R*_M_ calculations for LR glass beams in LTB (*n*_b_ = 2, *K* = var).

At the same time, the FE calculations give evidence of two different variations in the slope of numerical trends. As long as the restraints are weak, compared to the beam configuration, an increase in critical moment is appreciable, but no marked variations are expected for the global LTB response of the member (Figure 18b). Rigid restraints, on the other hand, are associated with a fully rigid bracing system and a typical LTB deformed shape with null local displacements for the *n*_b_ restrained nodes (Figure 18c). The intermediate configurations, accordingly, are characterized by a moderate increase of the expected critical buckling moment, as well as by a global LTB deformed shape that presents a partial bracing effect for the regions of LR supports (Figure 18b). Parametric calculations were, thus, repeated for all the previously defined beam geometries, so as to assess the expected scatter due to the approximation induced by the simplified analytical calculations. The so-obtained results are proposed in Figure 19, grouped by beam geometry.

**Figure 20:**
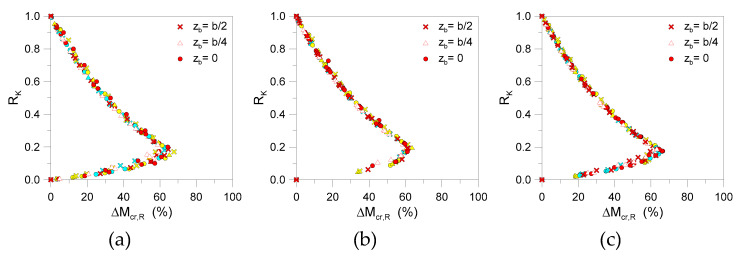
Percentage scatter (Equation (28)) for critical buckling moment estimates of selected LR glass beams (*n*_b_ = 2, *K* = var) grouped by span: (**a**) *L* = 2000 mm, (**b**) *L* = 3000 mm, and (**c**) *L* = 4000 mm (with *t* = 20, 30, 40 mm and *b* = 100, 200, 300 mm).

Differing from Section 5, a relatively high scatter was observed between the so-calculated critical buckling moments, especially in the presence of relatively weak restraints.In Figure 20, the scatter values given by Equation (28) are grouped by span *L* for the selected beams. As shown, the linearized approach underestimates, by up to 10–15%, the critical moment for *R*_K_ values higher than 0.7 (i.e., rigid restraints). The percentage scatter linearly increases (with up to a maximum of 60–70%) in the presence of relatively weak restraints (*R*_K_ = 0.1–0.2). Worthy of interest, in Figure 20, is the stable trend of the comparative dots, even for discrete restraints having different positions *z*_b_ on the beam height. In this regard, the linearized approach could still be used for LTB elastic calculations, as long as the general trend of Figure 20 is taken into account.

### 6.2. More Than Two Restraints (n_b_ = 3, 4, 5)

**Figure 21:**
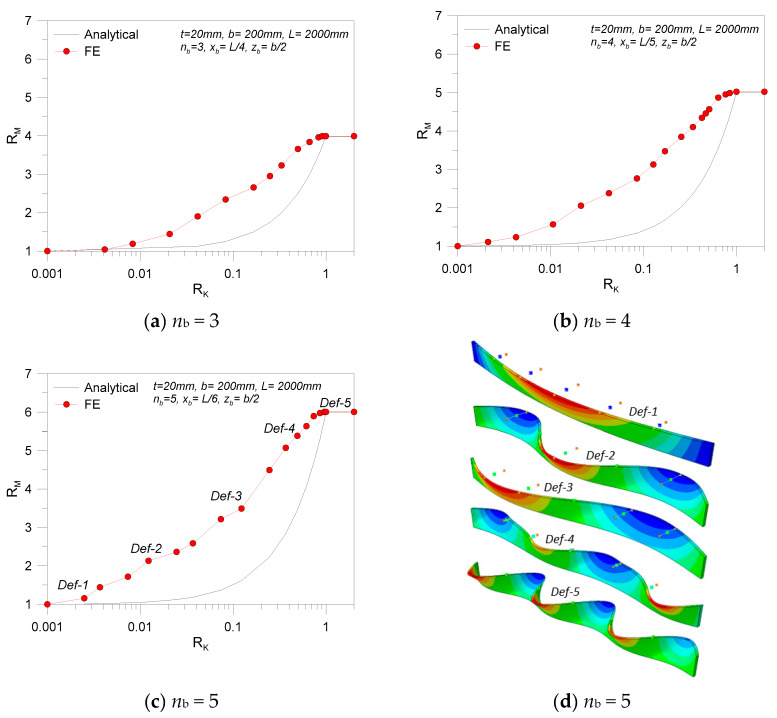
Comparative LTB calculations for LR glass beams with *n*_b_ > 1 discrete lateral restraints (with *K* = var): effects on (**a**–**c**) theoretical resistance and (**d**) selected critical deformed shapes (ABAQUS).

At the final stage of the study, the attention was focused on LR glass beams characterized by a relatively high number of restraints (up to *n*_b_ = 5, for the selected spans). In accordance with Figure 21, the parametric study gave evidence of relatively rough calculations from the linearized formulation.

Even in the presence of relatively stiff restraints (i.e., *R*_K_ > 0.7), the percentage scatter due to the analytical model was found on the order of 15%–20%, compared to FE estimates. Such a scatter (see Figure 21) was found to rapidly increase to 80%–90% for the numerical predictions for weak restrains (*R*_K_ ≈ 0.4), before progressively tending to 0 for extremely weak restraints (with negligible effects for LTB purposes). In parallel, the expected fundamental buckling shape was also found to be progressively modified, moving from the LTB response of a simple LU configuration (i.e., Figure 18d) toward a fully restrained deformed shape characterized by (*n*_b_ + 1) half-sine waves.

**Figure 22:**
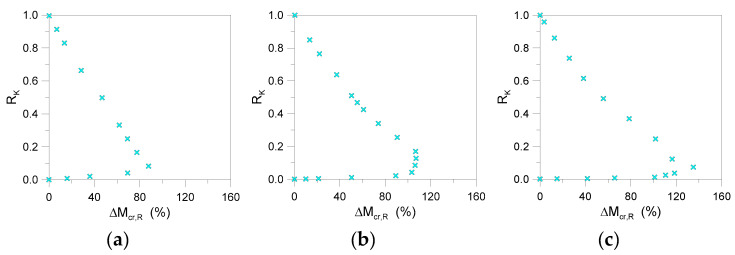
Percentage scatter (Equation (28)) for critical buckling moment estimates of selected LR glass beams (*K* = var): (**a**) *n*_b_ = 3, (**b**) *n*_b_ = 4, and (**c**) *n*_b_ = 5.

Among the selected configurations, Figure 22 shows that the presence of relatively weak restraints involves a progressively larger percentage scatter underestimation of critical buckling moments, compared to numerical methods. With respect to Figure 17; Figure 20 (where the maximum expected scatter was found in 8% and 70% of cases for glass beams with *n*_b_ = 1 or *n*_b_ = 2 weak restraints, respectively), such a percentage value increased to ~90%, ~115%, and ~150% for members characterized by *n*_b_ = 3, 4, or 5 discrete restraints. In the presence of stiff devices (i.e., *R*_K_ > 0.4), as shown in Figure 22, the calculated scatter variation is mostly linear with *R*_K_. Accordingly, the comparative results herein summarized could still offer a reliable feedback for simplified but still accurate critical buckling moment estimates on LR glass members in LTB.

### 6.3. Final Considerations

In conclusion, this research study highlighted some of the major issues that should be accounted for in the LTB analysis of structural glass members with discrete mechanical lateral restraints.

As long as the reliability of simplified analytical or numerical approaches that could be used for the calculation of the theoretical critical buckling resistance or LR glass beams are preliminarily addressed, it is important to further highlight that the same attention should be focused on the non-linear analysis of the same LR braced systems. As known, the knowledge of theoretical buckling loads is in fact a key step for design, but still poor information, compared to the actual load-bearing LTB response and resistance that LR glass members could offer. Major effects are expected, in this sense, from the shape and amplitude of initial geometrical imperfections to account for in non-linear LTB calculations. Moreover, another influencing parameter is expected to derive from the region of holes in glass members, where the stress peaks in LTB should be locally assessed, toward the overall validation of the standardized design approach recalled in Figure 3. Finally, the LTB response of LR glass members subjected to loading and boundary configurations differing from Figure 3 should also be separately investigated.

## 7. Conclusions

The assessment of the lateral–torsional buckling (LTB) behavior of structural glass members represents a key step for design, given the relatively high slenderness of load-bearing members that are typically used in constructions. In addition to such a design issue, most of the literature studies and design recommendations are available in documents and standards for laterally unrestrained (LU) glass members only, thus fully disregarding the effect (and potential) of discrete mechanical restraints that act as lateral bracing systems for laterally restrained (LR) glass members. In addition, an accurate estimation of the theoretical LTB critical moment for such LR systems represents a first key step for design. Often, however, the availability of simplified (but accurate) calculation approaches can offer robust support for designers.

In this paper, the attention was, thus, on the calculation of the elastic critical buckling moment of LR glass beams in LTB. A major advantage was taken from existing analytical approaches of the literature (even mostly developed for steel constructional members), as well as finite element (FE) numerical analyses, which were performed via the LTBeam tool and the ABAQUS computer software. 

Based on parametric calculations carried out on a wide series of geometrical and mechanical configurations of technical interest, the actual role and the effect of discrete mechanical restraints were explored for selected LR glass beams. Practical expressions were proposed to assess the expected stiffness contribution of selected mechanical point fixings in use for glass applications. In this regard, the reliability of simplified, spring-based numerical models was validated for selected configurations.

Moreover, the comparative analyses showed that the stiffness, number, and position of discrete mechanical restraints can have marked effects on the expected LTB estimates, with respect to traditional LU glass members. In addition, the use of a linearized analytical formulation (which was further adapted in this paper for LR glass beams) was found to offer rather good predictions, especially for members with single mechanical restraint (with a maximum 7–8% underestimation of theoretical critical moment). In the presence of two discrete restraints, the scatter of analytical estimates was observed to be still reliable for relatively stiff (or very weak, and thus negligible) restraints, while underestimates up to 60% were generally collected for intermediate stiffness configurations. Finally, the analysis of LR glass beams with multiple discrete restraints (up to five) typically resulted in even more pronounced scatter increases, as well as relatively high percentage errors (up 15–20%) for LR glass members with relatively stiff connectors. Furthermore, the trend of the so-calculated scatter was found to be relatively stable for several combinations of geometrical and mechanical parameters of technical interest. As such, the comparative studies herein summarized could represent useful feedback for practical LTB estimates.

## Figures and Tables

**Table 1:** Flexural equivalent section properties for laminated glass beams in LTB, according to [25].

	**2 glass layers**	**3 glass layers**
	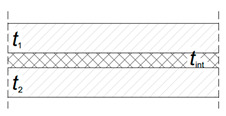	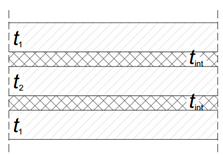
$t_{\mathrm{eq}}$	$\sqrt[{3}]{t_{1}^{3}+t_{2}^{3}+12\Gamma_{b}J_{s}}$	$\sqrt[{3}]{2t_{1}^{3}+t_{2}^{3}+12\Gamma_{b}J_{s}}$
$I_{z}$	$bt_{\mathrm{eq}}^{3}/12$	
$\Gamma_{b}$	$0\leq \frac{1}{1+\pi^{2}\frac{E\;t_{1}\;t_{2}\;t_{\mathrm{int}}}{\left(t_{1}+t_{2}\right)\;G_{\mathrm{int}}L^{2}}}\leq 1$	$0\leq \frac{1}{1+\pi^{2}\frac{E\;t_{1}t_{\mathrm{int}}}{4\;G_{\mathrm{int}}L^{2}}}\leq 1$
$J_{s}$	$t_{1}{\left(0.5t_{1}+0.5t_{\mathrm{int}}\right)}^{2}+t_{2}{\left(0.5t_{2}+0.5t_{\mathrm{int}}\right)}^{2}\;$	$t_{1}\;{\left(0.5t_{1}+0.5t_{2}+t_{\mathrm{int}}\right)}^{2}$

**Table 2:** Torsional equivalent section properties for laminated glass members, according to [25].

	**2 glass layers**	**3 glass layers**
	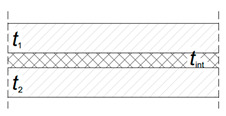	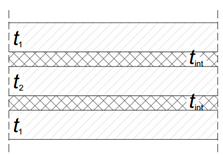
$I_{t}$	$I_{t,1}+I_{t,2}+I_{t,\mathrm{int}}$	$2I_{t,1}+I_{t,2}+I_{t,\mathrm{int}}$
$I_{t,i}$	$bt_{i}^{3}/3$ (i= 1, 2)	
$I_{t,\mathrm{int}}$	$J_{s,\mathrm{LT}}\;\left(1-\frac{\tanh\;\left(0.5\;\lambda_{\mathrm{LT}}\;b\right)}{0.5\;\lambda_{\mathrm{LT}}\;b}\right)$	
$J_{s,\mathrm{LT}}$	$4b\;\frac{t_{1}t_{2}}{t_{1}+t_{2}}{\left(0.5t_{1}+0.5t_{2}+t_{\mathrm{int}}\right)}^{2}$	$4b\;\frac{t_{1}t_{2}}{t_{1}+t_{2}}{\left(t_{1}+t_{2}+2t_{\mathrm{int}}\right)}^{2}$
$\lambda_{\mathrm{LT}}$	$\sqrt{\frac{G_{\mathrm{int}}}{G}\frac{t_{1}+t_{2}}{t_{1}t_{2}t_{\mathrm{int}}}}$	$\sqrt{\frac{G_{\mathrm{int}}}{G}\frac{2t_{1}+t_{2}}{4t_{1}t_{2}t_{\mathrm{int}}}}$

**Table 3:** Comparison of critical buckling moment variations for the selected beam geometry from Figure 11 (*n*_b_ = 1 and *n*_d_ = 2), as a function of *K* (3D = three-dimensional).

	M_cr,R_/M_cr,0_		
*K* (kN/m)	Analytical (Equation (19))	LTBeam (Spring-based Model)	FE (Model with 3D Restraints)
40	1.278	1.350	1.383
80	1.554	1.632	1.655
120	1.725	1.870	1.858
